# Loss of apoptosis-inducing factor critically affects MIA40 function

**DOI:** 10.1038/cddis.2015.170

**Published:** 2015-07-09

**Authors:** K Meyer, S Buettner, D Ghezzi, M Zeviani, D Bano, P Nicotera

**Affiliations:** 1 German Center for Neurodegenerative Diseases (DZNE), Bonn, Germany; 2 IRCCS Foundation Institute of Neurology ‘C. Besta', Milan, Italy; 3 MRC Mitochondrial Biology Unit, Cambridge, UK

## Abstract

Mitochondrial apoptosis-inducing factor (AIF) influences the oxidative phosphorylation (OXPHOS) system and can be recruited as a mediator of cell death. Pathogenic mutations in the *AIFM1* gene cause severe human diseases. Clinical manifestations include inherited peripheral neuropathies, prenatal cerebral abnormalities and progressive mitochondrial encephalomyopathies. In humans, rodents and invertebrates, AIF deficiency results in loss of respiratory complexes and, therefore, impaired OXPHOS. The molecular mechanisms underlying AIF-induced mitochondrial dysfunction remain elusive. Here we show that AIF physically interacts with the oxidoreductase CHCHD4/MIA40. In patient-derived fibroblasts as well as in tissues and glia cells from Harlequin (Hq) mutant mice, AIF deficiency correlates with decreased MIA40 protein levels, without affecting mRNA transcription. Importantly, MIA40 overexpression counteracts loss of respiratory subunits in Hq cells. Together, our findings suggest that MIA40 reduction contributes to the effects of AIF deficiency on OXPHOS, as it may impact on the correct assembly and maintenance of the respiratory subunits. This may be relevant for the development of new therapeutic approaches for AIF-related mitochondrial disorders.

Apoptosis-inducing factor (AIF) is an evolutionarily conserved mitochondrial protein initially described as a death effector.^1, 2^ The *AIFM1* gene maps to the X-chromosome and gives rise to a 67 kDa polypeptide that is imported into mitochondria in an unfolded form. The processed 62 kDa mature protein is mostly tethered to the mitochondrial inner membrane through a transmembrane domain, whereas a limited fraction is associated with the outer membrane.^3, 4^ As folded AIF incorporates flavin adenine dinucleotide (FAD) and possess nicotinamide adenine dinucleotide (NADH)-binding domains, it was initially proposed that AIF could function as low-turnover oxidoreductase. However, a significant number of recent biochemical data questioned this view and ruled out an antioxidant function, despite its potential redox properties.^5^ Although its enzymatic function remains unclear, AIF has emerged as a critical pro-survival housekeeping component of the mitochondrial oxidative phosphorylation (OXPHOS). In various cellular and animal models, AIF deficiency results in a general loss of respiratory elements, which shows some tissue specificity and is probably mediated by multiple factors, including altered expression, assembly and maintenance of the electron transport chain (ETC) subunits.^6, 7^ Although homozygous AIF-knockout mice are embryonic lethal,^8^ hypomorphic Harlequin (Hq) mutant mice are viable, although they display severe phenotypes.^9^ Hq mice express ~20% of normal AIF levels and exhibit reduced OXPHOS in various tissues. As a consequence of mitochondrial abnormalities, within 3–6 months of age Hq mice develop skeletal muscle atrophy, astrogliosis as well as progressive retinal and cerebellar neurodegeneration.^9, 10^ Recently, several pathogenic mutations in the *AIFM1* gene were identified in individuals exhibiting severe mitochondrial dysfunction, with a clinical spectrum that includes maternally inherited peripheral neuropathies, prenatal ventriculomegaly, fatal and slowly progressive encephalomyopathies and severe muscular atrophy.^11, 12, 13, 14, 15^ It remains to be defined whether the heterogeneity, onset and severity of these clinical manifestations are causally correlated with the extent of mitochondrial dysfunction. In general, mutations or a deletion of evolutionarily conserved amino acids result in significantly decreased AIF stability and, therefore, altered OXPHOS, although the underlying molecular mechanism remains unknown. Similarly to other mitochondrial disorders, clinical interventions are very limited.^16, 17, 18^

Here we searched for putative AIF interacting partners that might mediate OXPHOS deficiency. Our results show that mitochondrial intermembrane space import and assembly 40 (MIA40) co-immunoprecipitates with AIF. Importantly, MIA40 requires AIF for the physiological protein availability and function, resulting in an altered OXPHOS system in cells lacking AIF. Our findings unveil a novel pathway that may explain the loss of ETC subunits in individuals carrying AIF dysfunction, which may have significant implications for novel therapeutic approaches.

## Results

**Figure 1:**
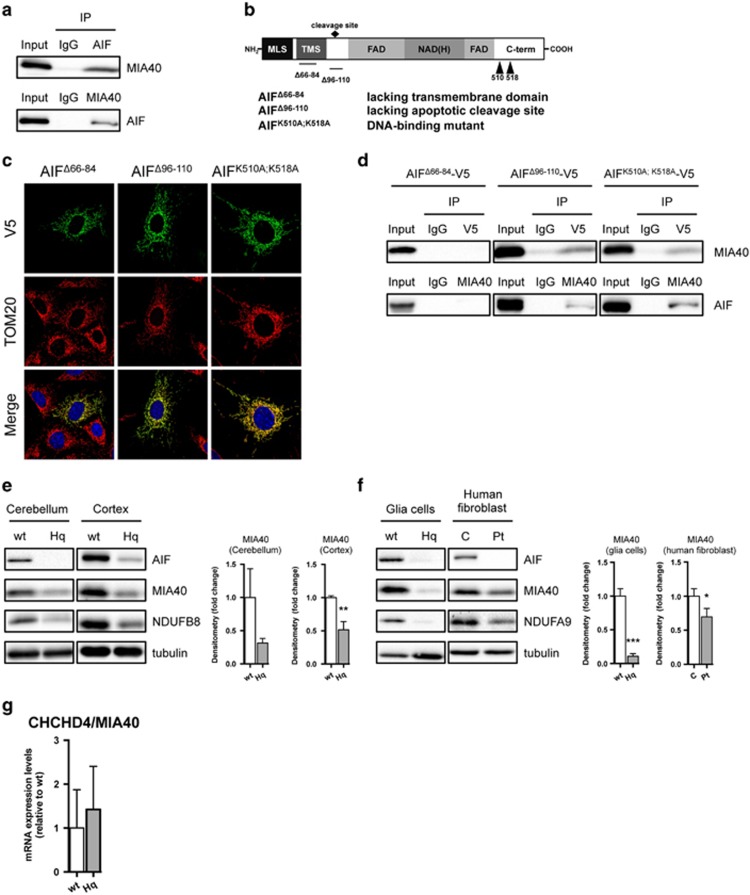
AIF interacts and regulates MIA40 protein. (**a**) Immunoblot analysis of AIF and MIA40 after immunoprecipitation (IP) with control IgG, AIF and MIA40 antibodies in HEK293T cells. (**b**) Illustration of AIF mutants lacking its transmembrane domain (AIF^Δ66-84^), its apoptotic cleavage site (AIF^Δ96-110^) or its DNA-binding capacity (AIF^K510A;K518A^). (**c**) Confocal imaging analysis of AIF mutants AIF^Δ66-84^-V5, AIF^Δ96-110^-V5 and AIF^K510A;K518A^-V5 using anti-V5 (green) and anti-Tom20 (red) antibodies. (**d**) Immunoblot analysis of AIF and MIA40 after IP with control IgG, V5 and MIA40 antibodies in HEK293T cells overexpressing AIF^Δ66-84^-V5, AIF^Δ96-110^-V5 and AIF^K510A;K518A^-V5. (**e** and **f**) Immunoblot and densitometry analysis of AIF, MIA40, NDUFB8 (CI), NDUFA9 (CI) and tubulin (as loading control) in cerebellum, cortex and glia cells derived from wild-type (wt) and Harlequin (Hq) mice as well as human fibroblast cells derived from control (**c**) and patients (Pt) carrying the *AIFM1* (R201Δ) deletion. (**g**) Quantitative RT-PCR analysis of CHCHD4/MIA40 in the cerebellum of 3 months old wild-type (wt) and Harlequin (Hq) mice. Values are plotted as means (+S.D.) and represent fold difference relative to control conditions. Asterisks show statistical significance (unpaired Student's *t*-test, *P<0.05, **P<0.01, ***P<0.001)

**Figure 2:**
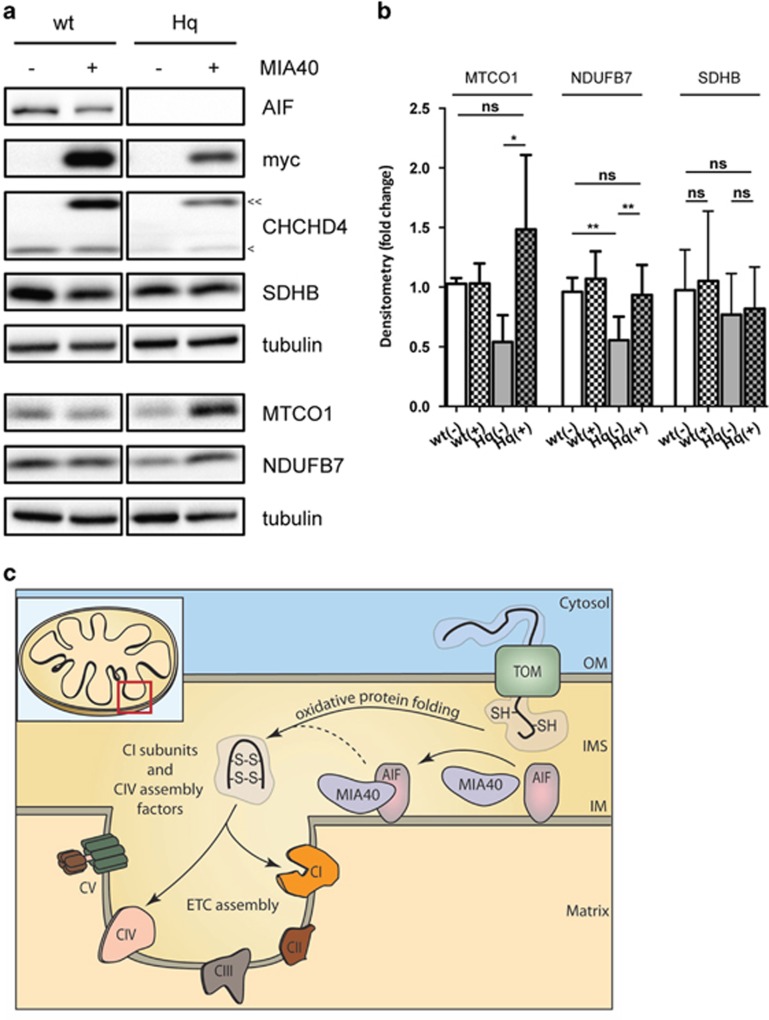
MIA40 overexpression ameliorates ETC deficiency in Hq MEFs. (**a** and **b**) Immunoblot and densitometry analysis of AIF, MIA40, NDUFB7 (CI), mitochondrially encoded cytochrome c oxidase I (CIV), SDHB (CII), CHCHD4/MIA40, myc-tagged CHCHD4/MIA40 and tubulin in wt and Hq MEFs transiently transfected with mitochondria-targeted green fluorescent protein (−) or MIA40-myc (+) plasmids. Endogenous CHCHD4/MIA40 (<) and myc-tagged CHCHD4/MIA40 (<<) are indicated. Values are plotted as means (+S.D.) and represent fold difference relative to control conditions. Asterisks show statistical significance (One-way ANOVA with Tukey's multiple comparisons test **P<0.01, *P<0.05, NS, non-significant). (**c**) Schematic representation of a simplified proposed working model. MIA40 substrates, such as CI subunits and CIV assembly factors are imported into mitochondria via the TOM complex. MIA40 binding to AIF results in efficient oxidative protein folding and ETC assembly

AIF deficiency significantly impairs OXPHOS in invertebrates, rodents and humans.^6, 9, 11, 12, 13, 19^ As AIF does not seem to interact with any ETC components,^5, 6, 20, 21^ we hypothesized that AIF influences OXPHOS maintenance through an interaction with a protein relevant for protein folding or ETC assembly. Thus, we used a yeast two-hybrid screening to identify AIF interacting partners with a contributing role in mitochondrial respiration. Using AIF as bait, we identified coiled-coil-helix-coiled-coil-helix domain containing 4 (CHCHD4)/MIA40 as a putative candidate with high confidence of interaction. MIA40 is a mitochondrial intermembrane space (IMS) protein that critically regulates the import and folding of small IMS and inner membrane proteins.^22^ Through a cascade of reversible redox reactions and interaction with augmenter of liver regeneration (ALR)/Erv1, MIA40 acts as a chaperone and contributes to the assembly of inner membrane complexes, including ETC components.^23, 24^ Notably, altered MIA40-Erv1 function causes an infantile mitochondrial disorder.^25^ To confirm the identified candidate from the yeast two-hybrid screen, we performed a co-immunoprecipitation assay in whole-cell lysates and, using an anti-AIF antibody, we showed that endogenous MIA40 interacted with AIF *in vitro* (Figure 1a). Similarly, endogenous AIF was co-immunoprecipitated using an anti-MIA40 antibody, confirming the physical binding (Figure 1a). To further assess the AIF-MIA40 binding, HEK293T cells were transiently transfected with green fluorescent protein-tagged AIF, however the yield was very low due to extensive cell death (data not shown). To overcome this limitation, we generated mutant AIF constructs with decreased apoptogenic action ^5, 26^ (Figure 1b). Specifically, V5-tagged AIF lacking the N-terminal transmembrane domain, the apoptotic cleavage sequence and the DNA-binding sites were individually transfected to cells. The three AIF mutant proteins localized correctly to the mitochondria (Figure 1c). Using an anti-V5 antibody, we performed immunoprecipitation assays in cellular lysates and found that the transmembrane domain comprising residues 66–86 is critical for AIF binding to MIA40 (Figure 1d). Given the described physical interaction between the two intermembrane proteins, we reasoned that AIF loss might affect whole MIA40 abundance. In line with this hypothesis, a significant decrease of MIA40 protein levels was found in brain tissue and glia cells derived from Hq mice as well as in human fibroblasts carrying the pathogenic *AIFM1* (R201Δ) deletion^12^ (Figures 1e and f). This effect appeared to be posttranscriptional, given that quantitative RT-PCR of MIA40 messenger RNA (mRNA) levels showed no significant change in the cerebellum of Hq mice compared with littermate controls (Figure 1g). As MIA40 is involved in the import and folding of both complex I subunits and complex IV assembly factors,^23, 24^ we hypothesized that overexpression of MIA40 might rescue some of the ETC defects in AIF-deficient cells. In line with our hypothesis, overexpression of MIA40 in Hq mouse embryonic fibroblasts (MEFs) led to a partial rescue of the complex I subunit NADH dehydrogenase (ubiquinone) 1 beta subcomplex (NDUFB7) as well as the complex IV subunit mitochondrially encoded cytochrome c oxidase I (MTCO1) at the protein level (Figures 2a and b). On the basis of these findings, AIF-mediated MIA40 stability is a critical process required for the proper folding and maintenance of the respiratory complexes (Figure 2c).

## Discussion

Mitochondria are critical regulators of cellular growth, proliferation and death. They significantly contribute to energy homeostasis by generating ATP through a series of redox reactions generally known as OXPHOS. The OXPHOS system comprises four respiratory chain complexes, two mobile electron carriers and the ATP synthase, which converts inorganic phosphate and ADP to ATP. A functional OXPHOS system requires a large number of molecular chaperones that control the proper assembly of over 90 individual proteins into soluble and membrane-embedded multisubunit complexes. Inherited mutations that affect mitochondrial bioenergetics and function significantly contribute to the onset of several human pathologies. In recent years, many pathogenic mutations in the *AIFM1* gene have been causally linked to disorders exhibiting abnormal mitochondrial bioenergetics. Although the role of AIF in cell death has been widely documented,^5, 27^ little is known about the mechanism through which AIF can affect the respiratory capacity of the cell. As the identification of the Hq mutation,^9^ it soon became evident that AIF has a fundamental role in survival. The following studies in cellular models confirmed that AIF deficiency primarily alters mitochondrial activity.^6^ As AIF has a NADH- and two FAD-binding domains, it possesses an oxidorductase activity and was, therefore, thought to act as an oxidant scavenger, protecting the respiratory complexes from locally generated reactive oxygen species. However, more recent biochemical evidence questions this hypothesis,^5, 28^ thereby leaving the molecular mechanisms underlying AIF-mediated mitochondrial deficiency unexplained. Here we show for the first time that AIF influences the stability of MIA40, which is a crucial component of the IMS-import and assembly machinery of cysteine-rich proteins.^22^ Such proteins are maintained in a reduced form in the cytosol and undergo both import and oxidative protein folding upon entry into the IMS in a MIA40-dependent manner. The oxidative folding mechanism involves a disulfide relay system in which electrons are shuttled from the entering substrate to MIA40, to the sulfhydryl oxidase Erv1/ALR and to a final electron acceptor, such as cytochrome c. Among MIA40 substrates are several complex I (CI) subunits and complex IV (CIV) assembly factors. As demonstrated in tissues as well as in cells, AIF deficiency correlates with a significant loss of MIA40 at the protein level. Importantly, MIA40 overexpression is sufficient to rescue the loss of ETC subunits in Hq cells. On the basis of our working hypothesis (Figure 2c), we postulate that one of the AIF physiological functions is to modulate the abundance as well as the function of the MIA40 protein. Theoretically, AIF may operate as a docking protein, recruiting factor or redox intermediate for MIA40. Reduced MIA40 functionality might result in an impaired import or oxidative folding capacity, leading to a reduced abundance of its substrates, such as CI subunits and CIV assembly factors. Therefore, the molecular binding between AIF and MIA40 would influence the correct assembly and maintenance of the ETC. Our observations unveil a novel mechanism by which AIF deficiency causes OXPHOS impairment. This may be of relevance in pathologies associated with mutations in the *AIFM1* gene.

## Materials and Methods

### Antibodies

The following primary antibodies were used in this study: rabbit-monoclonal AIF (Cell Signaling, Beverly, MA, USA); rabbit-polyclonal CHCHD4/MIA40 and rabbit-polyclonal NDUFB7 (Proteintech, Chicago, IL, USA); mouse-monoclonal V5, NDUFB8, NDUFA9, SDHB and total rodent OXPHOS antibody cocktail (Abcam, Cambridge, UK); mouse-monoclonal tubulin (Sigma-Aldrich, Munich, Germany); rabbit-polyclonal TOM20 (Santa Cruz, Santa Cruz Biotechnology, Dallas, TX, USA). Goat anti-rabbit and anti-mouse HRP-conjugated secondary antibodies were from Promega (Madison, WI, USA) and Pierce (Darmstadt, Germany), respectively. Goat anti-mouse Alexa Fluor 488 and anti-rabbit Alexa Fluor 568 were from Life Technologies (Darmstadt, Germany).

### Cell culture and transfection

HEK293T cells, human patient fibroblast, MEFs and glia cells were maintained in Dulbecco's modified Eagle's medium supplemented with 10% fetal bovine serum (Gibco, Darmstadt, Germany) and 1% penicillin–streptomycin (Gibco). Cells were incubated at 37 °C in a humidified incubator with 5% CO_2_. HEK293T cells were transfected using a calcium phosphate-based method. Human fibroblasts were transfected using Lipofectamin 3000 (Life Technologies). Lipofectamine was incubated in Opti-MEM for 10 min at room temperature, after which plasmid-DNA was added. The DNA-Lipofectamine solution was then incubated for 40 min at room temperature on a shaking wheel and finally added to cells, which were incubated in Opti-MEM (Life Technologies) at least 1 h before the transfection. Fresh media was added 2 h after transfection.

### Confocal analysis

Imaging analysis was performed using a Zeiss LSM 700 microscope (Zeiss, Oberkochen, Germany) with a 63 × immersion lens. Images were analysed and processed using Zen (Zen Software Ltd, Rochdale, UK) and ImageJ software. Before analysis, cells were cultured on cover slips, fixed for 5 min with 4% paraformaldehyde at 37 °C in a humidified incubator. Immunostaining was performed using primary anti-V5, anti-TOM20 antibodies and secondary Alexa Fluor 488 and Alexa Fluor 568 antibodies.

### Immunoprecipitation

For each immunoprecipitation 250 *μ*g of protein was used and incubated for 30 min at 4 °C with protein A-agarose (Sigma-Aldrich) to reduce unspecific binding. The supernatant was incubated with the primary antibody overnight at 4 °C. The next day the lysate was incubated with fresh protein A-agarose for 2 h at room temperature and afterwards the pellet was washed three times with RIPA buffer. Finally, the protein A-agarose pellet was resuspended in Laemmli-buffer, heated to 95 °C for 5 min and subjected to western blot analysis.

### Mitochondrial enrichment

Mitochondria were enriched before immunoprecipitation. Briefly, cells were collected and washed once with PBS. Then, the cell pellet was resuspended in IB buffer (10 mM Tris/MOPS, 1 mM EGTA/Tris, 2 mM sucrose, pH 7.4) and homogenized using a glass/teflon homogenizer. The homogenate was centrifuged 600 × *g* at 4 °C for 10 min. The supernatant was centrifuged and washed twice with IB buffer at 7000 × *g* at 4 °C for 10 min. The mitochondrial pellet was finally resuspended in RIPA buffer (Sigma-Aldrich), supplemented with protease and phosphatase inhibitor cocktail (Roche, Mannheim, Germany), sonicated and the protein concentration was determined by Bradford assay (Biorad, Hercules, CA, USA).

### Plasmids

The plasmid encoding CHCHD4-myc-DDK were obtained from OriGene (Rockville, MD, USA). The AIF^Δ96-110^-V5, AIF^K510A;K518A^-V5, AIF^Δ66-84^-V5 constructs in pLenti6.2-V5 were obtained from Life Technologies.

### Quantitative real-time PCR

RNA extraction, purification and reverse transcription were performed by using the QIAshredder, RNeasy RNA extraction kit (Qiagen, Hilden, Germany) and qScript cDNA SuperMix (Quanta Biosciences, Gaithersburg, MD, USA ). Quantitative RT-PCR was carried out in a Step One Plus Real time PCR System (Applied Biosystems, Darmstadt, Germany) and analyzed using the comparative ΔΔCt method. mRNA levels of beta-actin were used for normalization. The average of at least three technical repeats was used for each biological data point. The sequences of the oligonucleotides that were used are the following: mouse *beta-actin*: 5′-ggtggttcctccggaaagaa-3′ 5′-tgcgacattgatatccgtaagg-3′ mouse *CHCHD4/MIA40*: 5′-cgggaacaaccatgtcctac-3′ 5′-tcatggtcttctttggtcacaa-3′.

### SDS-PAGE and western blotting

Wild-type and Harlequin tissues as well as HEK293T, human patient fibroblast, MEFs and glia cells were lysed in RIPA buffer supplemented with protease and phosphatase inhibitors (Roche). Protein concentration was determined by Bradford assay and 20 *μ*g of protein was separated on a 12% SDS-acrylamide gel. Subsequently, the proteins were transferred onto a nitrocellulose membrane (Biorad), incubated with the desired primary antibody, followed by incubation with the appropriate secondary antibody. Chemiluminescent signals of enhanced detection substrates (Thermo Scientific, Darmstadt, Germany) were collected using a CCD camera-equipped ChemiDoc MP system (Biorad).

### Yeast two-hybrid analysis

Yeast two-hybrid screening was performed by Hybrigenics Services, S.A.S (Paris, France). The coding sequence for *Mus musculus Aifm1* (aa 102–612) (GenBank accession number GI:594150366) was PCR amplified and cloned into pB27 as a C-terminal fusion to LexA (N-LexA-Aifm1–C fusion). The entire insert was sequenced and used as a bait to screen a random-primed Mouse Adult Brain cDNA library constructed into pP6. Approximately 79.6 million clones were screened using a mating approach with YHGX13 (Y187 ade2-101::loxP-kanMX-loxP, mat*α*) and L40ΔGal4 (mata) yeast strains. On a medium lacking tryptophan, leucine and histidine, 302 His+ colonies were selected. The prey fragments of the positive clones were amplified by PCR and sequenced at their 5′ and 3′ junctions. The resulting sequences were used to identify the corresponding interacting proteins in the GenBank database (NCBI) using a fully automated procedure.

## Abbreviations

AIF: apoptosis-inducing factor
ALR: augmenter of liver regeneration
CHCHD4: coiled-coil-helix-coiled-coil-helix domain containing 4
CI: complex I
CIV: complex IV
ETC: electron transport chain
FAD: flavin adenine dinucleotide
IMS: intermembrane space
MEF: mouse embryonic fibroblast
MIA40: mitochondrial intermembrane space import and assembly 40
mRNA: messenger RNA
NADH: nicotinamide adenine dinucleotide
NDUFB7: NADH dehydrogenase (ubiquinone) 1 beta subcomplex
OXPHOS: oxidative phosphorylation
